# The Impact of the COVID-19 Pandemic on the Stroke Unit of a Portuguese Community Hospital

**DOI:** 10.7759/cureus.71851

**Published:** 2024-10-19

**Authors:** José Guilherme Assis, Ricardo Almendra, Andreia Matas, Andreia Veiga, Romeu Pires, Mário Rui Silva

**Affiliations:** 1 Department of Medicine, Centro Hospitalar de Trás-os-Montes e Alto Douro, Vila Real, PRT; 2 Department of Neurology, Centro Hospitalar de Trás-os-Montes e Alto Douro, Vila Real, PRT

**Keywords:** covid-19, stroke, stroke care, thrombectomy, thrombolysis

## Abstract

It is essential to understand the consequences of the COVID-19 pandemic on stroke care, contemplating the potential health and economic consequences of missed diagnoses or treatments. This study examined the pandemic's effects on stroke epidemiology, clinical characteristics, and treatment in a hospital setting, comparing data from March 2020 to February 2021 with the homologous period the previous year. A secondary analysis focused on the state of emergency periods (March 19 to May 2 and November 6 to February 28). The sample included 730 stroke admissions, with 343 during the pandemic. There was a 24.75% reduction in the average daily occupancy rate (p<0.001) and a slight decrease in average daily admissions, especially in June from 132% to 74% (p=0.01). There was a moderate negative correlation between the number of COVID-19 cases and occupancy rates in January (r{s}=-0.422, p=0.01) and February (r{s}=-0.532, p=0.01). Admissions for ischemic stroke decreased by 7.2% (p=0.026), particularly mild events, with a 6.8% decrease in lacunar syndromes (p=0.048). Mechanical thrombectomy (MT) increased by 5.4% (p=0.046), while intravenous thrombolysis (IVT) decreased by 2.8% (p=0.048), especially in severe stroke cases (-21.4%, p=0.001). The pandemic period documented more MT procedures than IVT. Hemorrhagic stroke admissions increased by 6.7% (p=0.01).

The analysis suggests a reduction in mild ischemic stroke admissions and fewer patients undergoing IVT for severe strokes during the pandemic. Broader-scale studies are necessary to confirm these findings. Considering the documented decline in diagnosis and treatment, minimizing the impact on stroke care during potential sanitary crises is paramount.

## Introduction

Stroke remains one of the main causes of morbidity and mortality in Portugal and in the world [1,2]. The timely administration of treatment in the acute phase, such as intravenous thrombolysis (IVT) and mechanical thrombectomy (MT), allows for the minimization of its impact, although requiring considerable healthcare organization [3].

In December 2019, the first cases of infection with the severe acute respiratory syndrome coronavirus-2 (SARS-CoV-2), later named coronavirus disease 2019 (COVID-19), were documented in Wuhan, China [4]. Due to the equilibrium between high transmissibility and low-to-moderate mortality, global spread is observed, later being declared a global pandemic by the World Health Organization [5]. On March 3, 2019, the first cases of COVID-19 were documented in Portugal, leading to the establishment of measures to contain the transmission of the virus, namely the enactment of the state of emergency and the confinement of the population [6]. According to the European Centre for Disease Prevention and Control, between March 2020 and February 2021, the average daily incidence of COVID-19 in Portugal fluctuated from 234 cases in the first considered month to 9,287 cases in January 2021. In Europe, during the same timeframe, reported values ranged from 133 cases in June 2020 to 5,752 cases in November 2020.

Since the beginning of the health crisis, the possibility of interference with the strategy for managing stroke has been raised [7]. In fact, some analyses show significant changes in profile attributed to the pandemic context, indicating a decrease in hospital admissions and employment of reperfusion therapies, with a reduction in patients undergoing IVT and MT [8-11]. Such variations may be related to the confinement measures imposed as well as the social consequences associated with the pandemic [12-16].

In this context, further research is crucial for understanding the COVID-19 pandemic's substantial impact on public health as a reflection of future health crises. The primary objective of this study was to delineate its effects on stroke care, particularly regarding admissions, clinical characteristics, and therapeutic approaches within hospital settings.

## Materials and methods

Cohort details

This retrospective observational study was based on a stroke unit (SU) level B, part of a tertiary hospital, located in Northern Portugal, which operated without resource limitations during the pandemic. The analysis took into account the hypothesis that the results of studies from other regions, or carried out on a global scale, may coincide with the Portuguese situation, reflecting a variation in the profiles of admission and acute management of cerebrovascular disease.

**Figure 1:**
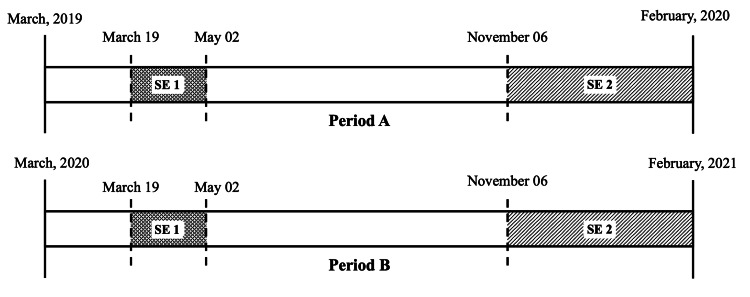
Follow-up and time intervals considered in the study. SE: state of emergency

The time interval for recruitment consisted of 24 months, divided into two periods of 12 months for comparative purposes of primary analysis - from March 2019 to February 2020 (period A) and from March 2020 to February 2021 (period B, corresponding to the pandemic). The protocols and stroke management guidelines implemented were identical between the two compared periods. In addition, subsequent analysis of time intervals with apparent relevance was established, including the state of emergency as well as the partial comparison between homologous months. The state of emergency was defined between March 19 and May 2 (state of emergency {SE} 1) and from November 6 to February 28 (SE 2) (Figure 1).

Inclusion and exclusion criteria

Data on individuals were gathered from the SU database and included all admissions observed during follow-up. The variables included general patient demographics, diagnosis (including stratification of ischemic stroke according to Bamford classification), performed treatment, hospital admission information (chronology and destination), and functional scales such as the modified Barthel and Rankin. The National Institutes of Health Stroke Scale (NIHSS) was collected, allowing the distribution of patients into four groups according to severity, namely, G1 (mild: 1-4), G2 (moderate: 5-14), G3 (severe: 15-24) and, G4 (very severe: ≥25). A composite of the mean number of vascular risk factors was calculated, which included diagnoses of hypertension (HT), atrial fibrillation (AF), diabetes mellitus (DM), dyslipidemia, obesity, and smoking. Information regarding the pandemic was extracted based on reports released by the Directorate-General of Health of Portugal [17].

Statistical analysis

Categorical variables were described through absolute frequency in numerals and/or relative frequency through percentages. Continuous variables were presented through the arithmetic mean and standard deviation if they followed a normal distribution, or median and interquartile range in the opposite case. Comparisons were established between a continuous and categorical variable using the Student’s t-test if normal distribution was followed, or the Mann-Whitney U test in the opposite case, and between two categorical variables through chi-squared test. In the appraisal of relationships between two continuous variables, the linear correlation was estimated as appropriate, and expressed by the Spearman coefficient. The composite of the mean number of vascular risk factors was calculated as the unit sum of the diagnoses considered for each patient (minimum 0 and maximum 6). The data were gathered in Microsoft Excel (Redmond, WA: Microsoft Corp.) and analyzed using IBM SPSS Statistics version 26 (Armonk, NY: IBM Corp.). The significance level was set at p<0.05.

## Results

The final sample comprised 730 admissions, mean age of 74.48±13.431 years, 48.8% of which were female. Between periods A (n=387) and B (n=343), there was an unequal proportion of female patients, 43.4% vs. 54.8% (p=0.002), with no identifiable significant difference in mean age. Regarding comorbidities, there was an increase in the prevalence of HT (p<0.001), AF (p=0.002), dyslipidemia (p<0.001), and obesity (p=0.001). Overall, the composite of the mean number of vascular risk factors per patient increased (p<0.001), including in SE 1 and SE 2 periods. The functional assessment scales applied, modified Barthel and Rankin scales, showed an increase in the degree of dependence on admission (p=0.629 and p=0.116, respectively) (Table 1).

**Table 1:** Demographic, clinical, and baseline characteristics for the patient cohort. *Hypertension, atrial fibrillation, diabetes mellitus, dyslipidemia, obesity, or smoking (minimum 0 and maximum 6). SE: state of emergency

Variables	Total	Period A	Period B	p-Value
Mean age (years), n	74.48	74.33	74.65	0.973
Female, n (%)	356 (48.8)	168 (43.4)	188 (54.8)	0.002
Number of patients in SE 1, n (%)	84 (11.5)	41 (10.6)	43 (12.5)	0.412
Number of patients in SE 2, n (%)	238 (32.6)	126 (32.6)	112 (32.7)	0.978
Mean number of vascular risk factors*, n	1.96	1.7	2.25	<0.001
Rankin scale on admission, n	3.37	3.33	3.41	0.629
Modified Barthel scale on admission, n	2.62	2.53	2.72	0.147
Number of patients, n (%)	730 (100)	387 (53.0)	343 (47.0)	-

**Figure 2:**
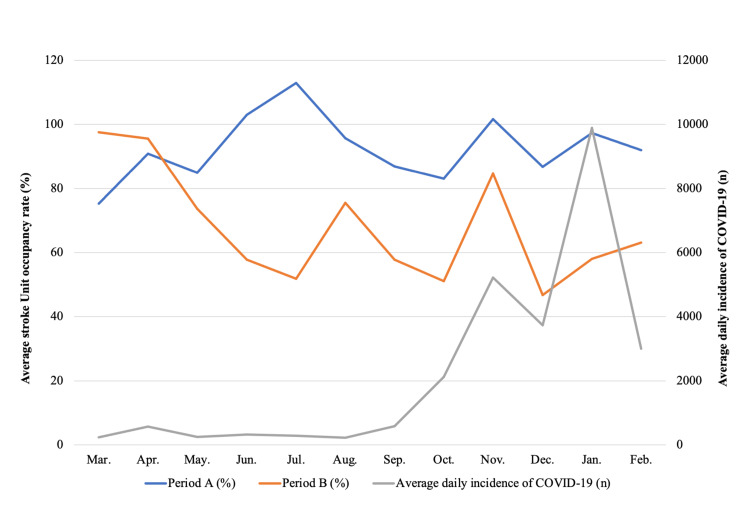
Average occupancy rate in periods A and B.

Comparing periods A and B, the average daily occupancy rate decreased from 92.5% to 67.8% (p<0.0001). In the monthly assessment, a significant difference is observed between June and February (p<0.001), reaching a maximum in December (86.83 vs. 46.77, p<0.001). However, this trend is exceptionally reversed in SE1, where an increase from period A to period B is noted (79.26% vs. 96.67%, p<0.001), primarily attributable to March (p<0.001) and April (p=0.265). Considering the pandemic interval, the increase in the number of confirmed new COVID-19 cases per day is associated with the decrease in the occupancy rate in January (r{s}=-0.422, p=0.01) and February (r{s} =-0.532, p=0.01) (Figure 2).

This relationship was not observed during the remaining time. The average number of daily admissions decreased from 1.05 to 0.94 (p=0.131), particularly in June, where it dropped from 1.32 to 0.74 (p=0.01). The average number of daily hospital discharges also decreased, from 1.06 to 0.93 (p=0.144), with a notable decline in June, from 1.52 to 0.71 (p=0.022). The average length of stay decreased from 9.86 to 7.27 days (p<0.001), especially in stroke G1, where it dropped from 5.660 to 4.236 days (p<0.001). In contrast, the mean interval between event and hospital admission increased from 1.15 to 1.45 days (p=0.562) (Table 2).

**Table 2:** Hospitalization metrics in the stroke unit.

Variables	Total	Period A	Period B	p-Value
Average occupancy rate, n (%)	9.62 (80.15)	11.10 (92.51)	8.13 (67.76)	<0.001
Admissions per day, n	1.00	1.05	0.94	0.131
Hospital discharges per day, n	1.00	1.06	0.93	0.144
Event-to-admission interval (days), n	1.38	1.15, n=87	1.45, n=339	0.562
Length of stay (days), n	8.64	9.86	7.27	<0.001
Number of patients, n (%)	730 (100)	387 (53.0)	343 (47.0)	-

Considering admissions for cerebrovascular events, the relative incidence of ischemic stroke decreased from 78% to 70.8% (p=0.026), while it increased in hemorrhagic stroke from 11.1% vs. 17.8% (p=0.01), and remained stable in transient ischemic attack. Regarding ischemic stroke, the proportion of lacunar syndromes decreased from 22.8% to 16% (p=0.048), while partial anterior circulation infarcts increased from 17.5% to 25.9% (p=0.018). The total anterior and posterior circulation infarcts did not show relevant variations. The NIHSS evaluation revealed an increase in the mean severity of events observed at admission, from 9.40 to 10.18 points (p=0.284). Further analysis revealed a decrease in G1, from 40.6% to 34.2% (p=0.134), and an increase in G2, from 32.2% to 37.6% (p=0.198), while G3 and G4 remained unchanged. The annual rate of IVT decreased from 10.6% to 7.8% (p=0.269). Conversely, the relative frequency of MT increased from 8.6% to 14% (p=0.046). According to severity, there is a decrease in IVT in G3, from 23.3% to 1.9% (p=0.001). The absolute number of MT was higher than IVT in period B (34 and 19, respectively), as opposed to period A (26 and 32, respectively) (Table 3).

**Table 3:** Differences in stroke according to the type of events. LACI: lacunar infarct; PACI: partial anterior circulation infarct; TACI: total anterior circulation infarct; POCI: posterior circulation infarcts; NIHSS: National Institutes of Health Stroke Scale; TIA: transient ischemic attack

Type of events	Total	Period A	Period B	p-Value
Ischemic stroke, n (%)	545 (74.7)	302 (78)	243 (70.8)	0.026
Bamford classification				
LACI, n (%)	108 (19.8)	69 (22.8)	39 (16)	0.048
PACI, n (%)	116 (21.3)	53 (17.5)	63 (25.9)	0.018
TACI, n (%)	205 (37.6)	111 (36.8)	94 (38.7)	0.644
POCI, n (%)	116 (21.3)	69 (22.8)	47 (19.3)	0.320
NIHSS				
G1 (0-4), n (%)	197 (37.7)	116 (40.6)	81 (34.2)	0.134
G2 (5-14), n (%)	181 (34.6)	92 (32.2)	89 (37.6)	0.198
G3 (11-24), n (%)	112 (21.4)	60 (21)	52 (21.9)	0.790
G4 (≥25), n (%)	33 (6.3)	18 (6.3)	15 (6.3)	0.987
Intravenous thrombolysis, n (%)	51 (9.4)	32 (10.6)	19 (7.8)	0.269
Mechanical thrombectomy, n (%)	60 (11)	26 (8.6)	34 (13)	0.046
Hemorrhagic stroke, n (%)	104 (14.2)	43 (11.1)	61 (17.8)	0.010
TIA, n (%)	35 (4.8)	18 (4.7)	17 (5)	0.847
Other diagnoses, n (%)	46 (6.3)	24 (6.2)	22 (6.4)	0.647
Number of patients, n (%)	730 (100)	387 (53.0)	343 (47.0)	-

During follow-up, an increase in mortality was observed, from 10.9% to 12.5% ​​(p=0.079), a variation that was maintained in SE1 and SE2 without reaching statistical significance. Duration of hospital stay prior to death decreased from period A to B, from 7.19 to 4.91 days (p=0.05). According to severity, mortality increased in G4, from 38.9% to 60% (p=0.027), with no significant variation in the remaining groups. In general, the number of patients enrolled in the National Integrated Continued Care Network (RNCCI) after discharge decreased, from 17.6% to 12.8% (p=0.076), as well as the time until placement, from 23.3 to 16.8 days (p=0.003).

## Discussion

During the pandemic, the average occupancy rate declined in the SU (-24.75%). At the beginning, in March, there is an isolated inaugural increase, which influences the SE1 period, corresponding to the first cases of COVID-19. Subsequently, contemporaneous with the increase in case incidence, the trend towards a reduction in the average occupancy rate develops in May and persists during the remaining examined period. In fact, in the months with the highest number of cases, January and February, the relationship between the number of cases and the average occupancy rate assumes a moderate negative correlation. As expected, the global average number of admissions and daily discharges decreased, confirming statistical significance only in June - the month with the highest absolute number of admissions and discharges in the previous year. The sample size likely limits the observed variability in admissions and discharges. The reduction observed in the length of stay reflects the decrease in the duration of hospitalization of less severe patients (G1), in a probable context of greater patient readiness for early discharge, reduction in the time until allocation of a vacancy in the RNCCI, increase in mortality in the most severe patients (G4), and earlier general in-hospital mortality.

The volume of admissions for ischemic stroke decreased (-7.2%), while hemorrhagic stroke increased (+6.7%). Initially, the general trend towards a decrease in the expression of less severe events is observed, attributed to the decrease in lacunar syndromes (-6.8%) and the tendency towards a decrease in mild stroke (G1), without reaching statistical significance. The reduction of less severe events - including mild, sensory, or minor motor deficits, with limited functional impact for the patient - ​​may be related to the devaluation of symptoms, leading to emergency department avoidance and underdiagnosis. On the contrary, the number of partial anterior circulation infarcts increased (+8.4%). Regarding the treatment, the relative number of patients undergoing MT increased (+5.4%), while IVT declined in severe stroke (-21.4%). The number of MT was higher than the number of IVT during the pandemic, contrary to the homologous period of the previous year. Given the different therapeutic windows, the variation observed in reperfusion therapies - specifically, the decrease in IVT (approved up to 4.5 h) and the increase in MT (indicated up to 24 h) - suggests a probable deferral in symptom reporting, possibly related to social factors caused by the pandemic, such as social isolation and fear of contact with the hospital.

Individuals admitted during the pandemic had a greater number of risk factors for vascular disease, namely, AH, AF, dyslipidemia, and obesity. Higher dependence on admission was not demonstrated. Variation in all-cause mortality was not proven; however, the present data suggest an increase in period B (additional research is needed to confirm this trend). The state of emergency did not significantly interfere with the analyzed variables.

This study suggests a change in the epidemiology of stroke in the context of a pandemic, confirming the decline in stroke metrics previously documented in other regions [15,16]. This reduction was not extended to hemorrhagic stroke and thrombectomy. Contrary to Portugal, many studies were based on global epicenters of the pandemic, with significant indirect damage from COVID-19 on health systems, which may justify the observed differences [7-15].

Due to the small dimension of this study, the sample size may constitute a limitation in obtaining significant results. The location of the center in Northern Portugal and the use of national data on the pandemic may interfere with the conclusions, as well as the extrapolation to other geographic areas in the country. Despite this, the confinements, measures enacted on a national scale, and social factors, transversal to the Portuguese reality, will not warrant further expected interference. The execution of larger-scale studies could be important to confirm this phenomenon. According to the SU admission criteria, patients with SARS-CoV-2 infection were excluded from this analysis. Given the growing evidence of the relationship between COVID-19 and thromboembolic events, its inclusion in subsequent studies will be pertinent [13]. The retrospective structure of the study represented a limitation in data collection, namely the event-admission interval, functional classification, and severity assessment.

Overall, this analysis provides data from the Portuguese context to the growing body of literature on the impact of the COVID-19 pandemic on stroke. It highlights the potential harmful consequences for both health and the economy resulting from the omission of stroke diagnosis or treatment.

## Conclusions

Ensuring access to and quality of stroke care is crucial. This study revealed significant changes in stroke admissions and management during the COVID-19 pandemic at a tertiary hospital in Northern Portugal. Specifically, there was a decline in average occupancy rates and admissions for ischemic stroke, particularly among milder cases. Additionally, a reduction in the number of patients receiving IVT for severe strokes indicates a negative impact on acute cerebrovascular care, amplifying the devastation caused by the pandemic on healthcare.

This research offers valuable insights into the COVID-19 pandemic's impact on stroke epidemiology and healthcare delivery. It underscores the need for further studies to explore the long-term consequences of these changes on patient outcomes and healthcare systems. Future research should involve larger, multicentric approaches to validate these findings and assess the direct effects of COVID-19 on stroke incidence and management. Investigating the psychosocial factors influencing patients' decisions to seek care during health crises will also be essential for improving emergency response strategies and ensuring timely intervention for stroke patients in future pandemics.
